# The impacts of the National Medication Price-Negotiated Policy on the financial burden of cancer patients in Shandong province, China: an interrupted time series analysis

**DOI:** 10.1186/s12889-022-14525-7

**Published:** 2022-12-16

**Authors:** Yi Ding, Chao Zheng, Xiaolin Wei, Qi Zhang, Qiang Sun

**Affiliations:** 1 Center for Health Management and Policy Research, School of Public Health, Cheeloo College of Medicine, Shandong University, Jinan, 250012 China; 2 NHC Key Lab of Health Economics and Policy Research (Shandong University), 44 Wen Hua Xi Road, 250012 Jinan, China; 3 Division of Clinical Public Health and Institute for Health Policy, Management and Evaluation, Dalla Lana School of Public Health, University of Toronto, Toronto, Ontario M5T3M7 Canada

**Keywords:** Price-negotiated policy, Anticancer medication, Financial burden, Interrupted time series analysis, China

## Abstract

**Background:**

In order to further regulate the price of anticancer medication and alleviate the financial burden of cancer patients, the Chinese government implemented the National Medication Price-Negotiated Policy (NMPNP) in 2017. This study aims to assess the impacts of implementation of the NMPNP on the access of anticancer medication and the financial burden for cancer patients in Shandong province, and to provide evidence to inform the design of similar policies in other developing countries.

**Methods:**

A quasi-experiment design of an interrupt time series analysis was conducted. The month of September 2017 was taken as the intervention point when the Shandong Provincial Reimbursement Drug Lists was updated based on the result of the NMPNP in 2017. The data used were the aggregated monthly claim data of cancer patients from 2016 to 2021, which were obtained from four cities in Shandong province. The outpatient and inpatient care visits per capita, proportion of OOP expenditure and medication costs in outpatient and inpatient medical costs were used as outcome variables. A segmented regression model was used to analyze the change of the access of anticancer medication and the financial burden for cancer patients.

**Results:**

The outpatient care visits per capita significantly decreased after the intervention. Compared to preintervention trend, the proportion of OOP expenditure in outpatient medical costs decreased by average 0.25 percentage point per month (*p* <  0.0001) after the intervention, however the proportion of OOP expenditure in inpatient medical costs increased by 0.02 percentage point per month (*p* = 0.76). Since the intervention, the proportion of medication costs in outpatient medical costs averagely rose by 0.28 percentage point (*p* <  0.0001), and its implementation caused the proportion of medication costs in inpatient medical costs averagely decreased 0.2 percentage point (*p* <  0.0001).

**Conclusions:**

The NMPNP improved the access of anticancer medication, and relieved the financial burden of outpatient care. However, it did not effectively alleviate the financial burden of inpatient care. Additionally, the NMPNP impacted the behavior of the healthcare providers. The policymakers should closely monitor the change of providers behaviors, and dynamically adjust financial incentives policies of healthcare providers during the implementation of similar medication price negotiated policies.

**Supplementary Information:**

The online version contains supplementary material available at 10.1186/s12889-022-14525-7.

## Background

Cancer is the one of noncommunicable diseases that threaten health and life of humans and is the one of the leading causes of death globally. In 2020, 19.3 million new cancer cases occurred worldwide, and the number of people who died from cancer nearly reached 10 million [1]. Previous studies have highlighted that cancer patients, especially in developing countries are undergoing high out-of-pocket (OOP) expenditure from cancer treatments [2]. WHO reported that the high price of essential anticancer medication including the targeted anticancer medication was the one of major reasons that aggravated the financial burden of cancer patients and their families [3]. Meanwhile, it also raises a series of major issues about the affordability and access worldwide [4]. Therefore, a few countries have designated healthcare authorities negotiate price of medication with pharmaceutical companies, so as to lower the price of costly anticancer medication and relieve the financial burden for cancer patients [5, 6].

As the biggest developing country in the world, in 2020, China accounted for 24.0% of new cancer cases and 30.2% of cancer-related deaths globally [7]. Furthermore, several researches have demonstrated that cancer patients and their families in China have suffered excessive financial burden and OOP expenditure from treatments. Such OOP expenditure of cancer patients even reached 63.8% of their annual household income; in particular, cancer patients among the low-income population spent nearly 88.0% of their annual household income on treatments [8]. Therefore, in order to further improve the affordability and access of anticancer medication, the Chinese government, as the biggest purchaser in the healthcare-sector, initiated the National Medication Price-Negotiated Policy (NMPNP) for anticancer medication in 2017, which aimed to lower the price of anticancer medication in the National Reimbursement Drug Lists (NRDL) through negotiations with the pharmaceutical companies [9]. Recent studies revealed that after implementing NMPNP in 2017, costs per defined daily dose (DDD) of targeted anticancer medication equally decreased by 48.9% and the expenditure of hospital medication decreased by 6.9% compared to pre-implementation [10].

However, there has been little empirical research examined whether NMPNP can effectively reduce OOP expenditure and consequently, relieve the financial burden of cancer patients in China. Therefore, in this study, we used the cancer patients as the sample population and also used a quasi-experimental design combined with interrupted time series analysis (ITS) to assess whether the NMPNP could effectively reduce the financial burden on cancer patients.

## Methods

### The National Medication Price-Negotiated Policy

In order to further regulate the price of anticancer medication and alleviate the financial burden of cancer patients, the Chinese government launched the NMPNP for anticancer medication in 2017. The Ministry of Human Resources and Social Security of the People’s Republic of China organized experts involved many related medical majors, such clinical medicine, pharmacy, pharmaceutical economics, and medical insurance, to formulate expected prices though evaluating the therapeutic effects, consumption of treatments and market price of anticancer medications. Subsequently, based on the expected prices, the experts negotiated the prices, types and payment standards of anticancer medications with pharmaceutical companies. If the negotiation was successful, the anticancer medications would be permitted into the NRDL, and the final prices of anticancer medications were regarded as the uniform payment standard throughout the country. In 2017, 18 anticancer medications covering lung cancer, stomach cancer, breast cancer, colorectal cancer, lymphoma, and myeloma were permitted in NRDL through negotiations, and the average procurement price of anticancer medication was reduced by 44% [11]. Since the National Healthcare Security Administration was established, the Chinese government successively permitted a total of 39 anticancer medications to go into NRDL by NMPNP from 2018 to 2019, and the average procurement price of anticancer medication continued to drop by 65% [12]. Until 2022, there were 76 anticancer medications in the NRDL [13].

Moreover, as a national compulsory policy, the National Healthcare Security Administration required the price of anticancer medication in all the Provincial Reimbursement Drug Lists (PRDL) must be updated according to the annual negotiated results within confined time, and public hospitals throughout the country were required to purchase those medications at the negotiated price [10]. In this case, in September 2017, the Shandong’s PRDL was updated the price of anticancer medications based on the negotiated results of NMPNP 2017, which was intended to alleviate the financial burden of cancer patients and increase the access of anticancer medication as soon as possible.

### Study design

As one of the provinces that has a large population, Shandong province has 7.2% of the Chinese population and has 7.33% of China’s gross domestic product (GDP) [14]. Additionally, the morbidities of gastric cancer, thyroid cancer, and breast cancer are separately higher than other provinces in China [15]. So, we took the implementation of NMPNP in 2017 in Shandong province as a quasi-experiment and took September 2017 as the intervention point in this study when Shandong’s PRDL was updated based on the negotiated results of NMPNP 2017. We also used an ITS design, which covers the complete claim data records of cancer patients from 2016 to 2021.

### Data sources

As the basic medical insurance that covers the largest insured population in China, Urban and Rural Resident Basic Medical Insurance (URRBMI) covers 74.03% of the Chinese basic medical insureds [16] including elders, children, and low-income rural and urban area residents [17]. According to the population and economic levels rank [18], we purposively sampled four cities in Shandong province (The sampling principle and data sources are shown in Additional file 1). We collected and aggregated the monthly medical claim data of 45,895 cancer patients who enrolled in URRBMI from the municipal healthcare security administrations of the four cities. The medical claim data of cancer patients are the related outpatient and inpatient medical costs of cancer treatments from January 2016 to December 2021. It contains healthcare service utilizations of outpatient and inpatient care, medical costs of outpatient and inpatient care, OOP expenditure of outpatient and inpatient care, and medication costs of outpatient and inpatient care.

### Outcome measures

In order to accurately quantify the access and affordability of anticancer medication, we assessed several outcome variables during study observation including outpatient and inpatient care visits per capita (Eq. 1), proportion of OOP expenditure in outpatient and inpatient medical costs (Eq. 2), and proportion of medication costs in outpatient and inpatient medical costs (Eq. 3). Regarded the China consumer price index (CPI) in 2016 as the base year, all data in the study was discounted by the CPI [14]. The skewed distribution of data in this study was log-transformed.

1 $$The\ outpatient/ inpatient\ care\ visits\ per\ capita=\frac{number\ of\ outpatient/ inpatient\ care\ visits\kern0.75em }{number\ of\ outpatient/ inpatient\ patients}$$

2 $$The\ proportion\ of\ OOP\ expenditure\ in\ medical\ costs=\frac{total\ OOP\ expenditure\kern0.75em }{ total\ medical\ costs}\times 100\%$$

3 $$The\ proportion\ of\ medication\ costs\ in\ medical\ costs=\frac{total\ medication\ costs\kern0.75em }{total\ medical\ costs}\times 100\%$$

### Statistical model and analysis

We used the ITS analysis to assess the change of access and affordability of anticancer medication in this study. The new itsa command contains the two ordinary least squares (OLS) regression in the Stata packages prais and newey, which can perform the ITS analysis for multiple inventions with single or multiple groups. In our ITS analysis, a segmented OLS regression model with a Newey-West test was separately used to assess whether NMPNP can reduce the financial burden of outpatient and inpatient care [19]. The segmented regression model we adopted is shown below:

$$Y_t=\beta_0+\beta_1T_t+\beta_2X_t+\beta_3T_tX_t+\varepsilon_t$$

*Y*_*t*_ is the dependent variable we measured at every monthly point *t*, *T*_*t*_ is the time series variable representing the time in months since the start of observation until to the time *t*, *X*_*t*_ is a dummy variable representing the intervention point (preintervention period is 0, post-intervention period is 1), *T*_*t*_ *X*_*t*_ is an interaction term of the time and intervention, and *ε*_*t*_ is the residual term representing the unknown variation component of the regression model. *β*_*0*_ represents the baseline level; *β*_*1*_ represents the baseline trend prior to intervention; *β*_*2*_ represents the immediate level change after the intervention compared to the preintervention; *β*_*3*_ represents the trend change after the intervention compared to the preintervention; and *β*_1_+ *β*_*3*_ represents the trend after the intervention [20].

The actest command was used to test the autocorrelation of time series [21], and the autocorrelation results were both present at lag 1. Due to the time trend of proportion of OOP expenditure in outpatient and inpatient medical costs in each month displayed conspicuous seasonal effect, and thus we used OLS regression model to perform the seasonality adjustment [22]. STATA 16.0 was used to perform all statistical analysis. Two-sided *P* <  0.05 was considered statistically significant.

## Results

### The basic information of cancer patients in the four cities

Table 1 demonstrates the basic information of cancer patients in the four cities from 2016 to 2021.

**Table 1:** Basic information of cancer patients in the four cities from 2016 to 2021

City	2016	2017	2018	2019	2020	2021
**City I**						
**Number of cancer patients in URRBMI** ^**a**^	**1880**	**2404**	**3010**	**4058**	**5014**	**5586**
**Gender**						
Male	584(31.06)	727(30.24)	941(31.26)	1418(34.94)	1801(35.92)	2117(37.90)
**Age groups (years)**						
≥ 45 ~ 60	726(38.62)	885(36.81)	1017(33.79)	1288(31.74)	1490(29.72)	1685(30.16)
≥ 60	1029(54.73)	1355(56.36)	1801(59.83)	2550(62.84)	3287(65.56)	3664(65.59)
**City II**						
**Number of cancer patients in URRBMI** ^**a**^	**4826**	**6087**	**8273**	**10,624**	**12,135**	**13,633**
**Gender**						
Male	2165(44.86)	2764(45.41)	3851(46.55)	4886(45.99)	5598(46.13)	6279(46.06)
**Age groups (years)**						
≥ 45 ~ 60	1833(37.98)	2251(36.98)	2855(34.51)	3551(33.42)	3969(32.71)	4326(31.73)
≥ 60	2527(52.36)	3352(55.07)	4832(58.41)	6407(60.31)	7479(61.63)	8622(63.24)
**City III**						
**Number of cancer patients in URRBMI** ^**a**^	**7550**	**9046**	**10,955**	**13,968**	**16,407**	**19,818**
**Gender**						
Male	2941(38.95)	3497(38.66)	4303(39.28)	5543(39.68)	6666(40.63)	8007(40.40)
**Age groups (years)**						
≥ 45 ~ 60	3042(40.29)	3458(38.23)	4039(36.87)	4939(35.36)	5611(34.20)	6830(34.46)
≥ 60	3827(50.69)	4828(53.37)	6105(55.73)	8111(58.07)	9795(59.70)	11,775(59.42)
**City IV**						
**Number of cancer patients in URRBMI** ^**a**^	**5131**	**3899**	**3660**	**7560**	**8879**	**9558**
**Gender**						
Male	2288(44.59)	1683(43.16)	1550(42.35)	3218(42.57)	3454(38.90)	3546(37.10)
**Age groups (years)**						
≥ 45 ~ 60	1688(32.90)	1277(32.75)	1183(32.32)	2425(32.08)	2802(31.56)	3113(32.57)
≥ 60	3026(58.97)	2349(60.25)	2239(61.17)	4644(61.43)	5535(62.34)	5879(61.51)

^**a**^ Urban and Rural Resident Basic Medical Insurance; The data are presented as n (%)

### The impacts of NMPNP on the healthcare service utilization of cancer patients

**Fig. 1:**
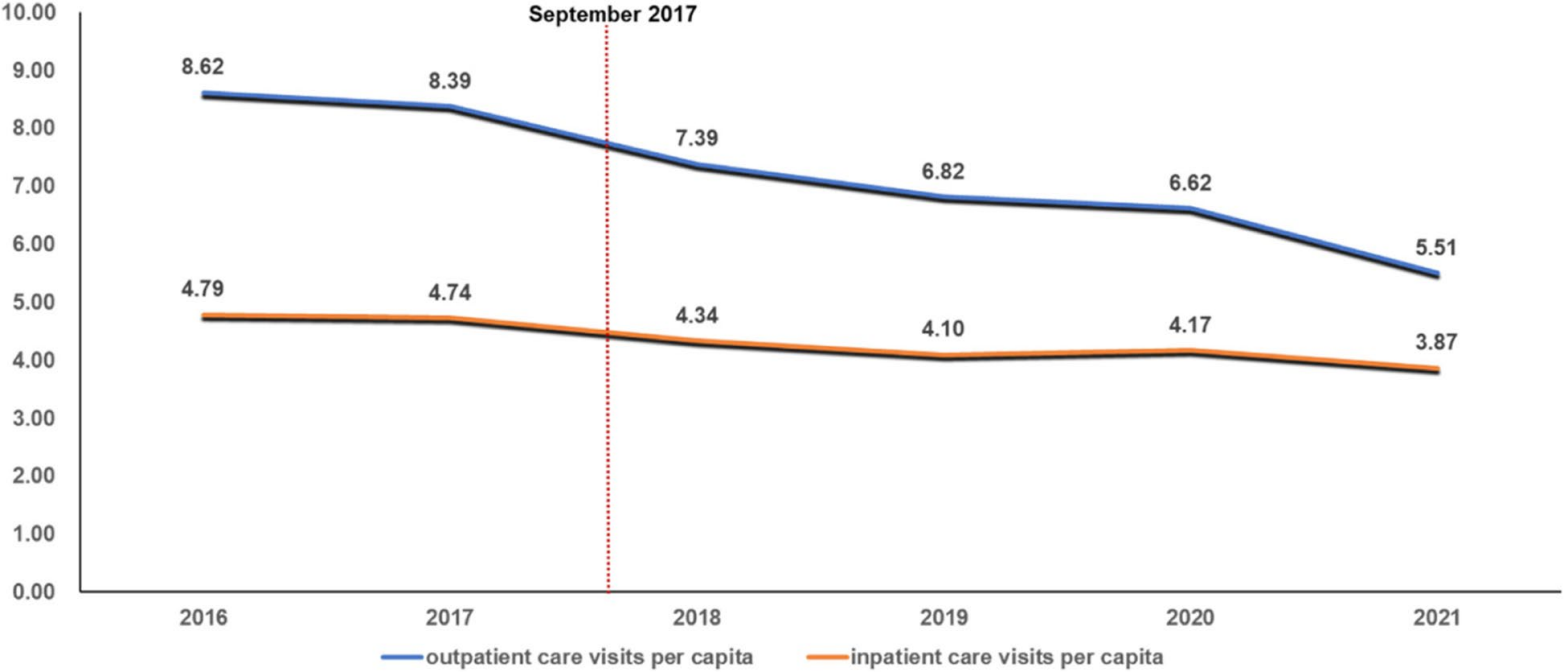
The impacts of NMPNP on the healthcare service utilization of cancer patients

Figure 1 demonstrates that the outpatient care visits per capita significantly decreased after the intervention, and the inpatient care visits per capita slightly decreased.

### The impacts of NMPNP on the financial burden of cancer patients

**Fig. 2:**
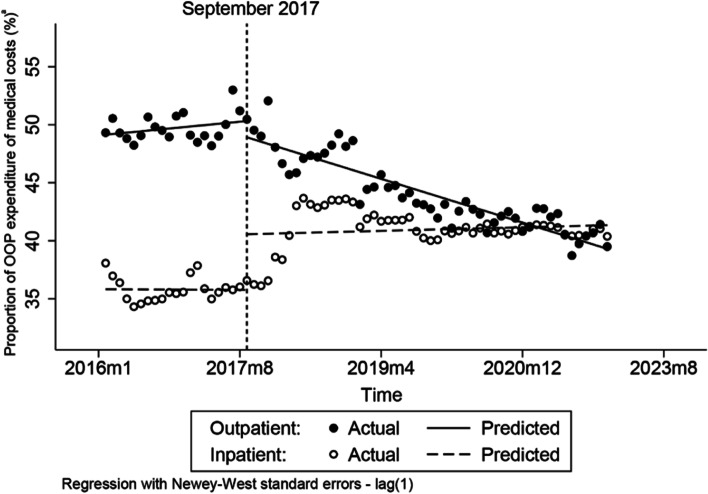
The impacts of NMPNP on the proportion of OOP expenditure in outpatient and inpatient costs. ^a^The proportion of out-of-pocket expenditure

Figure 2 demonstrates that the proportion of OOP expenditure in outpatient medical costs significantly decreased after the intervention; however, the proportion of OOP expenditure in inpatient medical costs significantly rose. After the intervention, the proportion of OOP expenditure in outpatient medical costs decreased 1.4 percentage points at the first month (*p* = 0.15) (Table 2), and then continued decreasing an average by 0.25 percentage point per month compared to the preintervention trend (*p* <  0.0001) (Table 2). However, the proportion of OOP expenditure in inpatient medical costs was immediately increased by 4.80 percentage point after the intervention (*p* <  0.0001) (Table 2) and then continued increasing by 0.02 percentage point per month compared to preintervention trend (*p* = 0.76) (Table 2).

**Table 2:** The change of levels and trends of outcome variables before and after intervention

Outcome variables	Outpatient			Inpatient		
	Coefficient	P	[95% Conf. Interval]	Coefficient	P	[95% Conf. Interval]
**Proportion of OOP expenditure (%)**^**a**^						
*β*_*0*_	49.14	< 0.0001	[48.24, 50.05]	35.82	< 0.0001	[34.4, 37.25]
*β*_*1*_	0.06	0.32	[−0.06, 0.18]	0.00	0.96	[− 0.11, 0.10]
*β*_*2*_	−1.40	0.15	[−3.34, 0.53]	4.80	< 0.0001	[2.48, 7.12]
*β*_*3*_	−0.25	< 0.0001	[− 0.37, − 0.13]	0.02	0.76	[− 0.1, 0.14]
*β*_*1*_ *+ β*_*3*_	− 0.19	< 0.0001	[− 0.22, − 0.16]	0.02	0.60	[− 0.04, 0.07]
**Proportion of medication costs (%)**						
*β*_*0*_	75.95	< 0.0001	[74.68, 77.22]	47.09	< 0.0001	[44.8 0, 49.38]
*β*_*1*_	−0.19	0.0050	[− 0.32, − 0.06]	−0.08	0.68	[−0.46, 0.30]
*β*_*2*_	−4.81	0.042	[−9.45, −0.18]	−7.35	0.018	[−13.41, −1.29]
*β*_*3*_	0.47	< 0.0001	[0.29, 0.65]	−0.12	0.53	[−0.50, 0.26]
*β*_*1*_ *+ β*_*3*_	0.28	< 0.0001	[0.16, 0.41]	−0.20	< 0.0001	[−0.22, − 0.17]

^a^The proportion of out-of-pocket expenditure

### The impacts of NMPNP on medication utilization of cancer patients

**Fig. 3:**
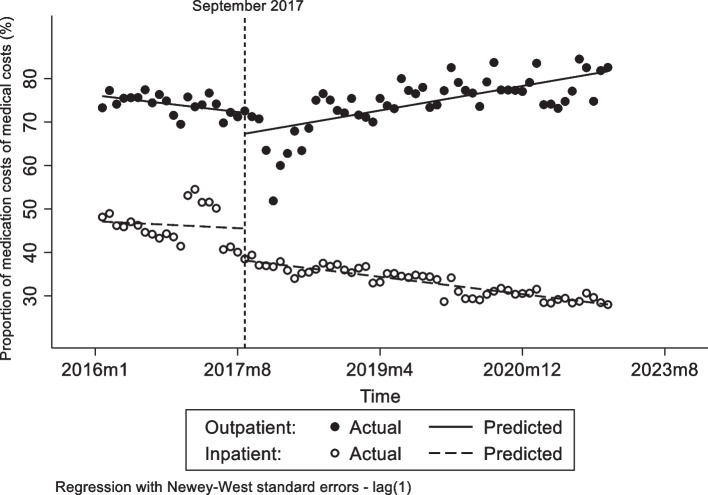
The impacts of NMPNP on the proportion of medication costs in outpatient and inpatient medical costs

With the NMPNP implemented, Fig. 3 demonstrates that the proportion of medication costs in outpatient medical costs rose and the proportion of medication costs in inpatient medical costs decreased. Although the proportion of medication costs in outpatient medical costs immediately decreased 4.81 percentage points after the intervention, however it subsequently rose with an increasing trend of 0.28 percentage point per month (*p* <  0.0001) (Table 2). Conversely, after the intervention, the proportion of medication costs in inpatient medical costs immediately decreased 7.35 percentage points (*p* = 0.018) (Table 2), and the post-intervention trend of proportion of medication costs in inpatient medical costs was reduced by 0.2 percentage point monthly compared to the preintervention trend as well (*p* <  0.0001) (Table 2).

## Discussion

After the NMPNP was implemented, we found that the outpatient care visits per capita of cancer patients was significantly decreased. Furthermore, the proportion of OOP expenditure and medication costs in outpatient costs displayed an obvious increase trend after the intervention, however these two outcomes in inpatient costs were opposite.

After the intervention, we found that the outpatient care visits per capita of cancer patients was decreased, however the proportion of medication costs in outpatient medical costs were increased. Based on that, we think the implementation of NMPNP effectively increase the access of anticancer medications in outpatient setting. Since 2017, more anticancer medications were brought into the NRDL though the implementation of NMPNP. With the development of the targeted anticancer medication, cancer patients who can use targeted anticancer medication can accept medication treatment in outpatient setting rather than using the inpatient care [23]. In this case, the implementation of the NMPNP increase the access of anticancer medication, while it further decreases the unnecessary outpatient care utilization of cancer patients. In addition, most of the cancer patients in this study entitled the Outpatient Special Chronic Diseases Policy (OSCDP), which is a unique policy that compensates for the outpatient medical costs of cancer patients. Therefore, under the situation that the reimbursement policies of outpatient care remained unchanged, the price reduction of targeted anticancer medication further released the healthcare need of those cancer patients to the targeted anticancer medication in outpatient while alleviating the financial burden of outpatient care.

However, what was unexpected was that the proportion of OOP expenditure in inpatient medical costs noticeably increased compared to before the NMPNP was implemented. The previous studies have underlined that medication policy reforms could impact the behavior of healthcare providers in China and have caused the rising of medication costs, medical consumables and OOP expenditure after the medication reforms were implemented [24]. In Fig. 3, the change of proportion of medication costs in inpatient medical costs clearly illustrates that the composition of inpatient medical costs was changed after the NMPNP was implemented, and giving us reasons to conclude that the NMPNP impacted the behavior of healthcare providers. In 2019, the Chinese government launched the zero mark-up drug policy (ZMDP) to public hospitals throughout China [25]. The implementation of ZMDP meant that the drug sales no longer was the major profit resource of public hospitals and conversely became the medical costs of public hospitals, which further weighted the pressure of costs-control of public hospitals. Accordingly, the revenue of public hospitals were principally dependent on the premiums of medical insurance after ZMDP implemented, and the public hospitals had to decrease the utilization of the drug listed in the NRDL for controlling the medical costs [26]. Furthermore, Fig. 3 illustrates that the NMPNP implementation led to the spillover effect of medication utilization of cancer patients. Hence, to the public hospitals in China, it is no doubt that the price reduction of anticancer medication due to the NMPNP ulteriorly increases the costs. Under that situation, hospitals had to curtail the volume of anticancer medication or prescribe more medical examination services through the payment transform [27], and even induced patients to use more medication outside the NRDL for controlling their medical costs [28]. This explains why the proportion of OOP expenditure in inpatient medical costs was significantly rose after the NMPNP was implemented. Therefore, healthcare policy makers should focus the spillover effect of NMPNP in time, and further optimize the financial incentives policies of healthcare providers and related supervision systems to guarantee the rational behavior of healthcare providers during the implementation process of NMPNP.

Finally, we suggest that a few developing countries that do not have high economic levels and sufficient medical recourses should consider implementing the similar NMPNP for anticancer medication to improve the access and affordability of medications to patients. However, we have a few suggestions for healthcare policy makers in developing countries.

First, limited by low economic and insufficient medical resource, healthcare policy makers should clearly know what to buy and how to buy at the beginning of the implementation of NMPNP in order to improve maximum value of strategic purchasing of medical insurance funds. In the implementation process of NMPNP, healthcare policy makers should establish a dynamic adjustment system to decide which types of medications can be covered by the medical insurance though evaluating the multiple factors, such as the economic levels, morbidity, and medical resources, which can improve the allocation efficiency of medical insurance funds.

Second, the price change of healthcare services can significantly impact the service utilization behaviors of patients. Therefore, after the NMPNP is implemented, healthcare policy makers should termly adjust the reimbursement policies according to the price change of negotiated medications. It can alleviate the financial burden of patients while preventing the moral hazard behaviors.

Third, we emphatically remind that healthcare policy makers should closely monitor the change of behavior of healthcare providers during the implementation process of NMPNP. Based on the results in this study, we think that the decrease of price of medications may cause a series of risk aversion behaviors of healthcare providers. Therefore, we suggest that healthcare policy makers should dynamically adjust financial incentives policies in the implementation process of NMPNP. On the one hand, healthcare policy makers should calculate the rational payment standard combined with NMPNP to ensure the provision initiative of healthcare service in healthcare providers; on the other hand, healthcare policy makers can adjust or optimize the provider payment methods to discipline the behavior of healthcare providers to improve the implementation effect of NMPNP.

## Limitations

This study has several limitations that needed to be addressed: First, because the NMPNP is a mandated policy throughout China, we hardly found a matched control group in our study. Hence, the net effect of NMPNP for cancer patients cannot be unambiguously demonstrated. Second, the data we used in this study were the aggregated monthly medical costs, which meant that the impacts of NMPNP on the affordability of different types of anticancer medication cannot be accurately evaluated yet. In the next step, we will prepare to collect the detailed medical cost records of various sorts of cancer patients according to the clinical indications of anticancer medication in the NMPNP, which can accurately assess the impacts of NMPNP on the financial burden of cancer patients. Third, we only used the proportion of medication costs in medical costs to illustrate that the NMPNP affected the behavior of healthcare providers. Therefore, we will further evaluate the impacts of the NMPNP on the behavior of healthcare providers through collecting the detailed composition of medical costs of outpatient and inpatient care of cancer patients, which reflects the costs of all types of healthcare services, and we will investigate on a deeper level that the relationship between the behavior of healthcare providers and the financial burden of cancer patients after the NMPNP was implemented.

## Conclusions

The NMPNP for anticancer medication improved the access of anticancer medication to the cancer patients. However, it did not effectively alleviate the financial burden of inpatient care. In addition, the proportion of OOP expenditure in inpatient care was drastically increased after the NMPNP was implemented. We also suggest that a few developing countries that do not have high economic levels and sufficient medical recourses should consider implementing similar medication price-negotiated policies to improve the access and affordability of medications to patients. However, it is worth noting that the healthcare policy makers should dynamically adjust the reimbursement policy according to the price of the price-negotiated medications and further optimize the financial incentives policies of healthcare providers and related supervision systems to improve the implementation effect.

## Supplementary Information

**Additional file 1.**

## Data Availability

The data that support the findings of this study are available from Healthcare Security Administrations but restrictions apply to the availability of these data, which were used under license for the current study, and so are not publicly available. Data are however available from the authors upon reasonable request and with permission of Healthcare Security Administrations.

## Abbreviations

NMPNP: National Medication Price-Negotiated Policy
OOP: out-of-pocket
NRDL: National Reimbursement Drug Lists
PRDL: Provincial Reimbursement Drug Lists
URRBMI: Urban and Rural Resident Basic Medical Insurance
ITS: Interrupted time series analysis
GDP: China’s gross domestic product
CPI: China consumer price index
OLS: Ordinary least squares
ZMDP: Zero mark-up drug policy
