# Supplementary material for: The impacts of the National Medication Price-Negotiated Policy on the financial burden of cancer patients in Shandong province, China: an interrupted time series analysis
Source: BMC Public Health. 2022 Dec 16;22:2363. doi: 10.1186/s12889-022-14525-7 (PMC9756446; doi:10.1186/s12889-022-14525-7)
Supplement: Supplementary file 1 — Additional file 1. [file 12889_2022_14525_MOESM1_ESM.docx]

**Additional file 1**

The data in this study was came from the project: **The Effect Evaluation of the** **Outpatient Payment Methods of Urban and Rural Resident Basic Medical Insurance (URRBMI), Shandong province**, which was supported by the Shandong Provincial Healthcare Security Administration.

Considering the main types of outpatient payment methods of URRBMI throughout the province, the population and economic levels rank of cities, and the access of data, we purposively sampled four cities (Table s1).

| **Table s1 The general information of the four cities in 2021** | | | | |
| --- | --- | --- | --- | --- |
| **Variables** | **City Ⅰ** | **City Ⅱ** | **City Ⅲ** | **City Ⅳ** |
| **Resident population (million)** | 2.91 | 2.97 | 4.71 | 2.20 |
| **Gender** |  |  |  |  |
| Male | 1.47(50.47) | 1.51(50.85) | 2.37(50.22) | 1.11(50.40) |
| Female | 1.44(49.53) | 1.46(49.15) | 2.34(49.78) | 1.09(49.60) |
| **Age groups (years)** |  |  |  |  |
| ≤14 | 0.34(11.82) | 0.55(18.36) | 0.7(14.89) | 0.38(17.35) |
| ≥14~60 | 1.78(60.88) | 1.76(59.21) | 2.91(61.87) | 1.37(62.27) |
| ≥60 | 0.76(27.30) | 0.67(22.43) | 1.09(23.24) | 0.45(20.39) |
| **GDP ^a^ per capita (thousand CNY)** | 106.92 | 74.52 | 89.24 | 156.90 |
| **^a^** Gross Domestic Product; The data are presented as n (%)  The access evaluation of healthcare services in outpatient setting was a part of our project. Meanwhile, as one of chronic disease which has heavy financial burden and can be treated in the outpatient settings, we brought the cancer disease into the implementation effect of the outpatient payment method in our project. Therefore, according to the ICD-10 codes. we collected and aggregated 1,383,891 medical records diagnosed as “cancer” during January 2016 to December 2021 from the healthcare security administrations of the four cities, which covered 949,401 outpatient care records and 434,490 inpatient care records of 45,865 cancer patients who enrolled in Urban and Rural Resident Basic Medical Insurance (URRBMI).  What a coincident thing was that these medical records covered the pre-intervention period and intervention period of implementation of the National Medication Price-Negotiated Policy (NMPNP) in Shandong province. In this case, we used these medical records of cancer patients as the data sources in this study, and to assess the impacts of NMPNP on the access of anticancer medication and financial burden of cancer patients. | | | | |
